# Complete Genome Sequence of Chlamydia avium PV 4360/2, Isolated from a Feral Pigeon in Italy

**DOI:** 10.1128/MRA.01509-19

**Published:** 2020-04-16

**Authors:** Anna Maria Floriano, Sara Rigamonti, Francesco Comandatore, Erika Scaltriti, David Longbottom, Morag Livingstone, Karine Laroucau, Alessandra Gaffuri, Stefano Pongolini, Simone Magnino, Nadia Vicari

**Affiliations:** a Department of Biology and Biotechnology Lazzaro Spallanzani, University of Pavia, Pavia, Italy; b National Reference Laboratory for Animal Chlamydioses, Istituto Zooprofilattico Sperimentale della Lombardia e dell’Emilia Romagna (IZSLER), Pavia, Italy; c Sky Net UNIMI Platform–Pediatric Clinical Research Center Romeo ed Enrica Invernizzi, Dipartimento di Scienze Biomediche e Cliniche Luigi Sacco, Università di Milano, Milan, Italy; d Risk Analysis and Genomic Epidemiology Unit, Istituto Zooprofilattico Sperimentale della Lombardia e dell’Emilia Romagna (IZSLER), Parma, Italy; e Moredun Research Institute, Pentlands Science Park, Bush Loan, Edinburgh, United Kingdom; f Bacterial Zoonoses Unit, French Agency for Food, Environmental & Occupational Health Safety (Anses), Maisons-Alfort, France; g Istituto Zooprofilattico Sperimentale della Lombardia e dell’Emilia Romagna (IZSLER), Bergamo, Italy; University of Arizona

## Abstract

We report here the whole-genome sequence of a Chlamydia avium isolate recovered from a feral pigeon in 1999 in Italy. Only one complete genome of a *C. avium* strain has been published so far. Future comparative analyses could provide valuable insights on the genomic evolution of the pathogen.

## ANNOUNCEMENT

The *Chlamydia* genus encompasses host-restricted bacteria characterized by a two-phase developmental cycle consisting of the alternation of an extracellular form, the elementary body, and an intracellular form known as the reticulate body (1). The genus currently includes 12 validly published species (2), and among them, C. psittaci, C. gallinacea, and C. avium have been isolated from birds. *C. avium* was originally isolated in Germany and Italy from pigeons and parrots (3). Only two *C. avium* genome assemblies are currently available (*C. avium* strain 10DC88 [3]), and one of them is part of an unpublished comparative genomic project (GenBank accession no. ASM41773.2).

We sequenced and investigated the genome of the *C. avium* field isolate PV 4360/2, isolated in 1999 from a pool of intestinal and liver tissue from a pigeon found dead but with no other signs of disease in the city of Bergamo, Italy, with no evidence of lesions at necropsy (4). The isolate was stored at the Italian National Reference Laboratory for Chlamydioses and cultured on LLC-MK2 cells. DNA was extracted from purified elementary bodies (5) using a NucleoSpin tissue kit (Macherey-Nagel), and a genomic DNA library was prepared using an Illumina Nextera XT kit following the manufacturer’s instructions. Whole-genome sequencing (WGS) was then performed on an Illumina MiSeq platform with a paired-end 250-bp run producing 2,036,824 paired-end reads with an average length of 235.9 bp; the read quality was checked using FastQC v. 0.11.4 (6). The *Chlamydia* reads were separated from host/contaminant reads using the Blobology bioinformatic pipeline (7), selecting reads mapping to contigs with a GC content of 0.3 to 0.4 and log_10_ (coverage) of ≥2.5× (Bowtie 2 v. 2.2.6) (8). Reads were then assembled using SPAdes v. 3.10 (9). Genome finishing was then performed with PCR validation on putative joins detected with Bandage software v. 0.8.1 (10). Thus, we obtained the complete genome assembly of the isolate, comprising a circular chromosome of 1,040,639 bp and a circular plasmid of 7,640 bp. The genome and the plasmid assemblies were then annotated with RAST v. 2.0 (11). Default parameters were used unless otherwise specified. A total of 947 genes in the chromosome plus 8 in the plasmid were predicted.

The genome of strain PV 4360/2 (size, 1,041,169 bp; GC content, 36.9%) presents a total of 3,696 single-nucleotide polymorphisms (SNPs), aligned to the only other *C. avium* complete genome available (reference strain 10DC88, GenBank assembly no. ASM58387.1) by Mauve v. snapshot version 2015-02-13 (12). No evidence of any genomic rearrangements or indels was detected. Seven Pmps (type V autotransporter proteins that play a key role in virulence and are involved in immune evasion) (13, 14) were identified. The Trp system, an important virulence factor in *Chlamydia* species, was not detected, whereas the *bioBFDA* system (15) is identical to that in the strain 10DC88.

Interestingly, alignment of the plasmid sequences identified a genomic region of 414 nucleotides (nt) present in the PV4360/2 plasmid that was absent from the 10DC88 plasmid. This region encodes two virulence genes, *pGP4-D* and *pGP3-D*. The former is truncated, and the latter is absent from the plasmid of the 10DC88 strain. This could explain the difference in length between the two plasmids. Furthermore, we found that the virulence gene *pGP8-D* was annotated as two separate genes in the 10DC88 plasmid.

These differences in virulence gene content show that further genomic studies could provide important insights on the evolution of the virulence of *C. avium*.

### Data availability.

The raw reads and assemblies have been deposited in ENA under the accession no. PRJEB25740. The strain is available at www.ibvr.org.
